# Feasibility of 3D T1W sequences in contrast-material enhanced MR cisternography at 3T

**DOI:** 10.55730/1300-0144.5542

**Published:** 2022-09-12

**Authors:** Oktay ALGIN, Ural KOÇ, Gıyas AYBERK

**Affiliations:** 1 Department of Radiology, City Hospital, Bilkent, Ankara, Turkey; 2 Department of Radiology, Yıldırım Bayezid University, Ankara, Turkey; 3 National MR Research Center, Bilkent University, Ankara, Turkey; 4 Department of Neurosurgery, Yıldırım Bayezid University, Ankara, Turkey

**Keywords:** 3D-SPACE, 3D-VIBE, cisternography, 3-tesla, cerebrospinal fluid leakage

## Abstract

**Background/aim:**

We investigated the diagnostic values and artifact severities of 3D-T1W sequences in the diagnosis of cerebrospinal fluid (CSF) leakage.

**Material and methods:**

We retrospectively reviewed 3-tesla contrast-material enhanced MR cisternography exams of 22 patients with suspected CSF leakage in 4 years. The presence of the artifacts on 3D-T1W data was evaluated using a 4-point scale (0: none; 1: minimal; 2: moderate; 3: prominent). Agreements between CSF leakage results of the 3D-T1W sequences and consensus decisions were evaluated via kappa values. Artifact scores were analyzed by Fisher’s exact test.

**Results:**

The most compatible techniques with the consensus diagnoses were fat-saturated 3D-T1W-SPACE and 3D-T1W-VIBE sequences. The most artifact containing the 3D-T1W sequence was 3D-MPRAGE.

**Conclusions:**

3D-SPACE and 3D-VIBE are more successful in evaluating CSF leakages compared to 3D-MPRAGE. 3D-SPACE has lower artifact scores compared to 3D-VIBE and 3D-MPRAGE sequences.

## 1. Introduction

Contrast-material enhanced MR cisternography (CE-MRC) provides physiologic and morphologic details of the skull base without radiation, and it has quite a high accuracy for cerebrospinal fluid (CSF) leakages [1]. Routine 2D sequences have limited anatomical coverage for optimal CE-MRC [2]. Besides, total imaging times and slice thicknesses of multiplanar 2D-T1W acquisitions were generally higher than a single plane isotropic 3D-T1W acquisition [3]. 3D-T1W turbo spin-echo technique (also called 3D-SPACE or 3D sampling perfection and application-optimized contrasts using the different flip-angle evolutions) was recently introduced for high-field MR units since they offer high-resolution isotropic data with various image contrasts and low specific absorption rate values for the whole cranium within a short time [3]. Isotropic 3D-SPACE acquisitions facilitate multiplanar, oblique, and/or freehand curved reconstructions to better evaluate the contrast-media leakages and intracranial complex lesions/structures [4]. 3D-T1W-SPACE and 3D-VIBE sequences have higher contrast-to-noise ratios and visual conspicuity ratings compared with 3D-MPRAGE [5]. Detection of contrast material enhancement and visualization of lesion-enhanced lesions are more prominent in 3D-T1W-SPACE and 3D-VIBE compared to 3D-MPRAGE in the literature [5].

Generally, CE-MRC examinations are made in the prone position. Therefore, more motion artifacts are observed in CE-MRC images compared to routine MR exams obtained in the supine position. All isotropic 3D-T1W sequences still lack clinical validation for CE-MRC in patients with CSF leakages. In this retrospective study, we compared CSF leakage detection capabilities and artifact scores of three commercially available 3D T1W MRI sequences at 3-tesla (3T) in patients with suspected CSF leakage. According to our knowledge, there is no comparative study about the artifacts and diagnostic roles of 3D T1W MRI sequences at 3T in patients with suspected CSF leakage.

## 2. Materials and methods

According to respective institutional guidelines, an institutional review board approval was not required for this type of retrospective study. All the procedures being performed were part of the routine care. Informed consent was obtained from all subjects before the exams. In pediatric cases, informed consent was obtained from both parents and the patient. Patients who have (or suspected) otorhinorrhea and underwent 3T CE-MRC in 4 years period were included in this retrospective study. After a retrospective analysis of these patients and the CE-MRC images, 22 patients with CE-MRC obtained with 3D-T1W sequences were included in the study.

### 2.1. Inclusion criteria

Patients who have CSF leakage or otorhinorrhea suspicion based on neurosurgeon, otolaryngologist, or neurologist evalualtion, and patients with CE-MRC exam and 3T 3D-T1W (at least 3D-SPACE, 3D-VIBE, and 3D-MPRAGE) images were included in the study.

### 2.2. Exclusion criteria

Patients without detailed clinical and follow-up information were exluded from the study.

### 2.3. Acquisition details of MR exams

All CE-MRC exams were acquired on a 3T MRI unit (Magnetom Skyra; Siemens) with a 20-channel head coil. After obtaining noncontrast material-enhanced 3D-T1W and heavily 3D-T2W (noncontrast MR cisternography) images, lumbar puncture was performed by using a 22-gauge needle at the L3–L4 level under sterile conditions. Subsequently, 2 mL of saline mixed with 0.5 mL of gadobutrol was injected intrathecally for all patients. During transfer to the 3T MR unit, the patient is kept in bed in a prone position. Three hours after the gadobutrol injection, CE-MRC images were obtained in a prone position (if necessary, late phase postcontrast images were obtained). All patients were clinically observed in the hospital for 24 h after the procedure. Details of CE-MRC acquisitions are given in Table 1.

### 2.4. Evaluation of the data

MRC images were evaluated on a PACS workstation by two neuroradiologists (6 and 15 years of neuroradiology experience) that were blinded to clinical data using a Leonardo workstation (Siemens, Erlangen, Germany). Curved, freehand, or oblique multiplanar reconstructions and maximum intensity projection images were generated from 3D datasets. An extension of spontaneously hyperintense CSF on 3D heavily T2W images (for NCE-MRC) and/or hyperintense contrast-material-enhanced CSF on postcontrast 3D T1W images (for CE-MRC) from the cerebral cistern into the paranasal sinuses, nasal cavity, or middle ear cavity was regarded as the positive finding for CSF leakage for each sequence, as stated in the literature [6, 7]. Pre- and postcontrast CE-MRC images were analyzed together to avoid false-positive results.

The final or definite diagnosis of the presence of CSF leak was established according to the physical exam, clinical, beta-2-transferrin test, thin-section CT, CT cisternography, and CE-MRC findings. If present, surgical and endoscopic results were used to confirm the diagnosis. Any disagreement related to the final diagnosis was resolved by consensus of the authors. All cases were followed during regression of the symptoms after CE-MRC exams and surgical and endoscopic therapies to verify the CSF leakage presence/localization and complications.

Moreover, the presence of the artifacts on 3D-T1W data was evaluated using a 4-point scale (0: none; 1: minimal; 2: moderate; 3: prominent). Follow-up clinical exam and imaging data were used to evaluate changes after the intrathecal gadolinium administration.

### 2.5. Statistical analysis

Data were analyzed using SPSS v.21.0 software (SPSS IBM Inc., Chicago, IL, USA). Age and follow-up time related to the cases were given as mean, standard deviation, median, and minimum-maximum values. Agreement between techniques was evaluated via kappa values. Artifact scores of sequences were given as number and frequency. Artifact scores of the sequences were compared via Fisher’s exact test. Some 3D-T1W images or sequences of some patients were not available related to the PACS or hospital information system updates. These sequences were not included in the statistical analysis. p < 0.05 was accepted as statistically significant.

## 3. Results

**Figure 1:**
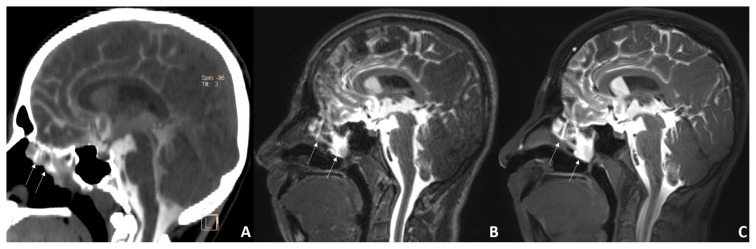
Sagittal CTC (A) and CE-MRC (B-C) images of a male with posttraumatic rhinorrhea history. In these images, CSF leakage towards the left ethmoid sinuses is observed (arrows). More artifacts are seen on the T1W 3D-MPRAGE (the middle) image than on the T1W 3D-SPACE (the right).

**Figure 2:**
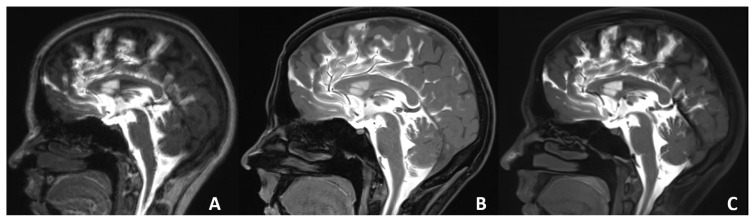
Sagittal T1W 3D-MPRAGE (A), 3D-VIBE (B), and 3D-SPACE (C) images of a 28-year-old female with posttraumatic skull base fractures and rhinorrhea history. There is no CSF leakage on the images. More artifacts are seen on the 3D-MPRAGE image than in 3D-VIBE and 3D-SPACE images.

**Figure 3:**
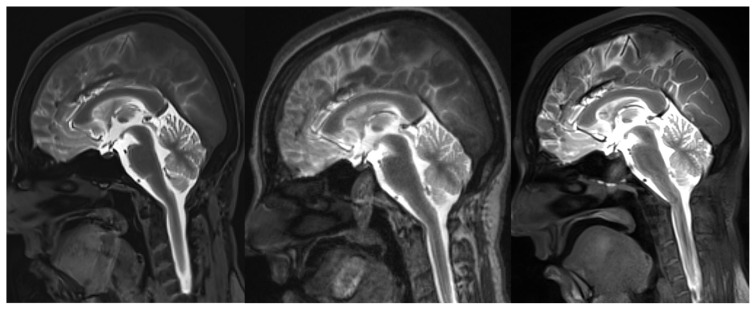
Sagittal postcontrast T1W 3D-VIBE (A), 3D-MPRAGE (B), and 3D-SPACE (C) images of a patient without CSF leakage. More artifacts are seen on the T1W 3D-MPRAGE image than on the 3D-SPACE and 3D-VIBE images.

**Figure 4:**
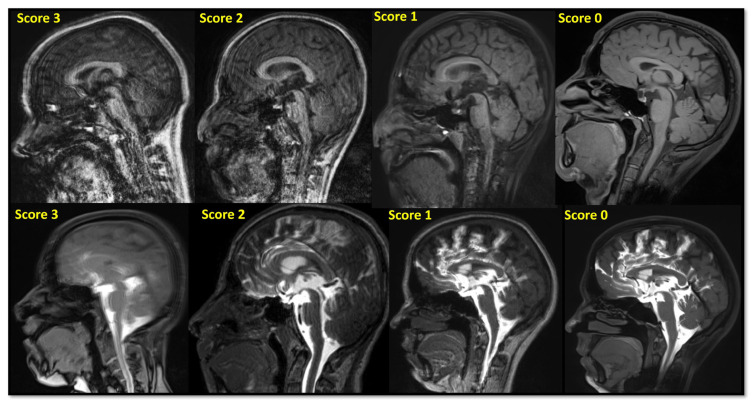
The artifact scores of the cases.

CSF leakage was not detected in 5 cases. The arachnoid cyst was detected in 2 cases. In 11 cases, there were also CTC examinations obtained in the same week with CE-MRC. Indication of CE-MRC exam was rhinorrhea in 19 cases and otorrhea in 3 cases. CSF leakage was detected in 17 cases based on consensus sessions (right in 6 cases, left in 11 cases). Eight of these had trauma history, and one of them had postoperative leakage. The most common leakage locations were cribriform plate (in 6 cases) and ethmoid sinuses (in 5 cases) (Figure 1). The ages and follow-up durations of the cases are given in Table 2. The most compatible techniques with the consensus or final diagnoses were fat-saturated 3D-T1-SPACE and 3D-T1-VIBE sequences (Table 3). The technique least compatible with the consensus scores was CTC (Table 3). Among the 3D-T1W sequences, the most artifact observed sequence was the 3D-MPRAGE (Figure 2, Table 4). There is a statistically significant difference between artifact scores of the 3D MPRAGE and T1-SPACE sequences according to Fisher’s exact test (p = 0.011) (Figure 3, Table 5). No statistically significant difference was detected in 3D-MPRAGE&3D-VIBE or 3D-VIBE&3D-T1-SPACE comparisons for artifact scores (Figure 4, Table 5).

All cases were followed for at least 3 years. No major complication was detected in the patients. Minor complications (postprocedural headache lasting less than 2 days or limited epidural hemorrhage) were observed in three patients. None of the patients with negative CE-MRC reports had a new finding or recurrence of rhinorrhea during this follow-up period.

## 4. Discussion

In patients with CSF leakage, determination of the leakage site in the preoperative period is crucial for increased surgical success rate and reducing the possible complications [2,7]. Although intrathecal gadolinium injection has not been confirmed yet by FDA, many short- and long-term studies show that low-dose (≤1 mL/mmol) intrathecal gadolinium administration is safe and more successful than the other imaging modalities (such as high-resolution CT or CTC), as observed in our study [1,2]. Our study shows the added values of fat-saturated T1W 3D-SPACE and 3D-VIBE sequences compared to 3D-MPRAGE, NCE-MRC, and/or CTC for the evaluation and diagnosis of CSF leaks.

3D-T1W data were relatively newly introduced at 3T. 3D-T1W sequences still lack validation for clinical use in patients with suspected CSF leakage. We observed that the multiplanar reconstruction capabilities of 3D-T1W sequences were very useful for the demonstration of CSF leaks in complex-shaped tiny regions.

Three-tesla isotropic 3D-T1W-SPACE has a shorter acquisition time and a larger coverage area (including the whole cranium) than multiplanar 2D-T1W spin-echo-based acquisitions [6]. Isotropic 3D imaging offers a global 3D view of the entire skull base, paranasal sinuses, meninges, and brain with high signal-to-noise and contrast-to-noise ratios [2]. Moreover, we find that 3D-T1W-SPACE is less susceptible to movement or susceptibility artifacts compared to other 3D-T1W techniques related to the turbo-spin-echo nature of the 3D-SPACE technique as mentioned in the literature [8]. According to our experiences, simultaneous use of 3D-T1W (especially 3D-SPACE and 3D-VIBE) sequences together increases the reporting comfort of the radiologist and combines the advantages of the techniques.

It was reported that 3D-T1W-SPACE and 3D-VIBE have higher contrast-to-noise ratio values and visual conspicuity compared with 3D-MPRAGE [5]. Subtly or small CSF leaks detected by 3D-T1W-SPACE and 3D-VIBE may be crucially missed by 3D-MPRAGE. Our results confirm and extend previous investigations conducted on 3D-T1W sequences for the evaluation of brain lesions [5].

This study has several limitations. Our first and most important limitation is the low number of study cases. The other limitation is the absence of surgical or beta-2-transferrin test results in all cases of CSF leakage. These limitations are also present in many studies about cisternography or CSF leakages [9]. Future larger studies are needed to assess the value of 3D-T1W-SPACE and 3D-VIBE for the evaluation of CSF leakages.

In conclusion, 3D-T1W-SPACE and 3D-VIBE are more successful in evaluating CSF leakages compared to 3D-MPRAGE. 3D-T1W-SPACE is less susceptible to movement or susceptibility artifacts compared to 3D-VIBE and 3D-MPRAGE.

## Figures and Tables

**Table 1:** The parameters of the 3-tesla MRI sequences.

Sequence parameters	3D-MPRAGE	T1W-3D-SPACE	T1W-3D-VIBE	HT2W-3D-SPACE	3D-CISS
**TR** (ms)	2300	600	4	3200	8.68
**TE** (ms)	2.38	11	1.43	411	4.03
**FOV** (mm)	210 × 100	250 × 91	240 × 100	240 × 100	150 × 100
**Average**	1	1	3	1	1
**Slice thickness** (mm)	0.82	0.96	1.1	0.94	0.47
**Fat saturation**	+/−	+	+	−	−
**Distance (gap)** %	50	−	20	−	20
**Voxel size** (mm)	0.8 × 0.8 × 0.8	0.96x.0.96×0.96	1.1 × 1.1 × 1.1	0.94x.0.94x.094	0.5x.0.5x.05
**Flip angle**	8°	120°	9°	120°	50°
**Inversion time** (ms)	900	NA	NA	NA	NA
**Number of slices**	192	144	144	160	64
**Phase oversampling** (%)	0	0	10	0	60
**Acquisition time** (min)	5.21	3.36	5.16	4.46	10.15
**PAT factor**	2	2	2	2	1
**Phase encoding direction**	A-P	A-P	A-P	A-P	A-P
**Slice oversampling** (%)	16.7	11.1	11.1	10	37.5
**Orientation**	Sagittal	Sagittal	Sagittal	Sagittal	Coronal or axial

**Abbreviations:** 3D: Three-dimensional; FOV: field of view; 3D-MPRAGE: 3D magnetization-prepared rapid gradient-echo, PAT: parallel imaging technique, NA: not applicable

**Table 2:** Mean, median, and min-max values of age and follow-up duration of the patients.

	Age	Follow-up duration
Mean	36.60	472.45
Median	31.00	105.50
Minimum	15.00	5.00
Maximum	77.00	2554.00
Std. deviation	17.29	735.58

**Table 3:** Correlation coefficient and Kappa values of consensus scores.

	Correlation coefficient	Kappa value	p-value	Agreement level
CT cisternography (CTC)	0.854	0.47	0.024	Moderate
MPRAGE	0.966	0.909	<0.001	Almost perfect
T1W-SPACE	1.000	1	<0.001	Almost perfect
T1W-VIBE	1.000	1	<0.001	Almost perfect
HT2W (NCE-MRC)	0.891	0.913	<0.001	Almost perfect

**Table 4:** Artifact scores of the sequences.

		Case number (n) (%)		
Sequences		MPRAGE	T1W-SPACE	T1W-VIBE
Scores	0	3 (20)	17 (81)	7 (58.3)
	1	7 (46.7)	3 (14)	4 (33.3)
	2	2 (13.3)	1 (5)	1 (8.3)
	3	3 (20)	0 (0)	0 (0)

**Note:** Data are case numbers with percentage in parenthesis.

**Table 5:** Comparisons between artifact scores of the sequences.

	p-value*
MPRAGE vs T1W-SPACE	0.011
MPRAGE vs T1W-VIBE	0.6
T1W-VIBE vs T1W-SPACE	0.061

* Fisher’s exact test
